# Possible Lingering Effects of Multiple Past Concussions

**DOI:** 10.1155/2012/316575

**Published:** 2012-02-26

**Authors:** Grant L. Iverson, Ruben J. Echemendia, Amanda K. LaMarre, Brian L. Brooks, Michael B. Gaetz

**Affiliations:** ^1^Department of Psychiatry, University of British Columbia, 2255 Wesbrook Mall, Vancouver, BC, Canada V6T 2A1; ^2^Research Department, Copeman Healthcare Centre, 400-1128 Hornby Street, Vancouver, BC, Canada V6Z 2L4; ^3^Psychological and Neurobehavioral Associates Inc., 204 East Calder Way, Suite 205, State College, PA 16801, USA; ^4^Memory and Aging Center, Department of Neurology, University of California, San Francisco, Suite 905, 350 Parnassus Avenue, San Francisco, CA 94143-1207, USA; ^5^Neurosciences Program, Alberta Children's Hospital, 2888 Shaganappi Trail NW, Calgary, AB, Canada T3B 6A8; ^6^Faculty of Medicine, University of Calgary, 2500 University Drive NW, Calgary, AB, Canada T2N 1N4; ^7^Department of Kinesiology and Physical Education, University of the Fraser Valley, Abbotsford, BC, Canada V2S 7M8

## Abstract

*Background*. The literature on lingering or “cumulative” effects of multiple concussions is mixed. The purpose of this study was to examine whether athletes with a history of three or more concussions perform more poorly on neuropsychological testing or report more subjective symptoms during a baseline, preseason evaluation. *Hypothesis*. Athletes reporting three or more past concussions would perform more poorly on preseason neurocognitive testing. *Study Design*. Case-control study. *Methods*. An archival database including 786 male athletes who underwent preseason testing with a computerized battery (ImPACT) was used to select the participants. Twenty-six athletes, between the ages of 17 and 22 with a history of three or more concussions, were identified. Athletes with no history of concussion were matched, in a case-control fashion, on age, education, self-reported ADHD, school, sport, and, when possible, playing position and self-reported academic problems. *Results*. The two groups were compared on the four neuropsychological composite scores from ImPACT using multivariate analysis of variance followed by univariate ANOVAs. MANOVA revealed no overall significant effect. Exploratory ANOVAs were conducted using Verbal Memory, Visual Memory, Reaction Time, Processing Speed, and Postconcussion Scale composite scores as dependent variables. There was a significant effect for only the Verbal Memory composite. *Conclusions*. Although inconclusive, the results suggest that some athletes with multiple concussions could have lingering memory deficits.

## 1. Introduction

Sport-related concussions result in temporary neurocognitive deficits and subjectively experienced physical, emotional, and cognitive symptoms in the initial hours, days, and sometimes weeks after injury [1–17]. In group studies, athletes tend to recover in terms of perceived symptoms and neuropsychological test performance within 2–28 days, with most studies suggesting recovery occurs in 5–10 days [7, 10, 13, 15–17]. There is some evidence suggesting that high school athletes might recover more slowly than university or professional athletes [18–21].

Athletes with prior concussions are at statistically increased risk for a future concussion [22–25]. Even more concerning is whether athletes with previous concussions are at risk for long-term damage to the structure and/or function of their brains. Numerous studies have been published employing cross-sectional methodologies in an attempt to determine whether groups of previously concussed athletes appear to have lingering effects detectable using symptom rating scales, neuropsychological testing, and electrophysiology. Literature on this subject is somewhat mixed, partly due to methodological differences and limitations across studies. Moreover, some aspects of the research designs and data analyses vary considerably across studies, and many researchers have not consistently reported effect sizes. Sample size is usually the limitation because the accrual of multiply concussed athletes is slow. For example, several published studies have sample sizes of multiply concussed athletes of fewer than 20 (e.g., [26–31]). Other studies do not define the injury severity characteristics of prior concussions (e.g., [9, 27, 32–40]) or when the prior concussions occurred (e.g., [9, 22, 27, 28, 30, 32, 34–38]). Most studies rely on a cross-sectional methodology of examining small [28, 29, 34, 41] or large [2, 22, 27, 32, 35, 38–40, 42] groups of athletes during baseline preseason testing. Some studies, however, have followed multiply concussed athletes prospectively [2, 22, 29, 32–34, 38, 43, 44].

Despite the methodological challenges associated with this area of research, there is an accumulation of evidence that some athletes with a history of multiple concussions might have lingering, long-lasting adverse effects. In a large scale NCAA study, Collins and colleagues [2] reported that athletes with a history of two or more concussions (i.e., 2–10) reported more symptoms and performed more poorly on two neuropsychological tests than athletes with no concussion history. Other studies have reported that athletes with a history of three or more concussions have changes in electrophysiology, [28, 31, 40] subjective symptoms, [28, 34] and neuropsychological test performance [34]. One large study involving jockeys (*N* = 618) reported that those with two or more previous concussions did not report more subjective symptoms or perform more poorly on neuropsychological testing than those with no previous concussion. When comparing jockeys with a history of one prior concussion to those with two or more prior concussions, those with more injuries performed more poorly on a single neuropsychological test (Stroop Color-Word Test) [39]. Stephens et al. [42] administered a battery of neuropsychological tests to a group of athletes and found that those with previous concussions performed more poorly on a test of attention and a visual memory test. In contrast, some large-scale studies have not found differences in neurocognitive functioning in multiply concussed athletes [27, 32, 45].

Due to concerns that the mixed results observed in the literature are due to differences in the type of neuropsychological tests employed, Bruce and Echemendia [46] investigated the relationship between self-reported history of concussion and neuropsychological test performance in a large multisport sample of college athletes using three testing approaches: (1) performance on a computerized neuropsychological test battery (ImPACT), (2) performance on traditional paper-pencil neurocognitive tests, and (3) the relationship between concussion history and performance on both traditional neuropsychological tests and computerized neuropsychological tests. None of the approaches yielded statistically significant relationship between history of multiple concussion and neurocognitive deficits. Finally, evaluations of mechanisms of cumulative subconcussive impacts have not shown a definitive link between repeated impacts such as exposure to heading a soccer ball [42, 47] or impact biomechanics in football to concussion [48]. Thus, recent studies have not clearly demonstrated whether a lingering effect of three or more previous concussions can be reliably detected. Moreover, given that most research designs are cross-sectional (not prospective), statements about causation must be made cautiously.

Given the contradictory findings, more research is needed. The purpose of this study was to examine whether athletes with a history of three or more concussions perform more poorly on neuropsychological testing, or report more subjective symptoms, during a baseline, preseason evaluation. A precise matching of athletes on numerous variables, in a case-control fashion, was used to improve the methodological rigor of the study.

## 2. Method

### 2.1. Participants

An archival database including 786 male athletes who underwent preseason testing with a computerized battery (ImPACT) was used to select the participants. Twenty-six athletes, between the ages of 17 and 22 (mean age = 19.7, SD = 1.4), with a self-reported history of three or more concussions, were identified (16 sustained three previous concussions, six four previous concussions, two five previous concussions, one six previous concussions, and one ten previous concussions). Athletes with no self-reported history of concussion were precisely matched, in a case-control fashion, on age (*M* = 19.7, SD = 1.4; *t*(50) = 0.04, *P* = .97; each person matched within 6–12 months of age) and education (*M* = 12.8, SD = 1.1; *t*(50) = 0.23, *P* = .82; each person matched within one year of education). Athletes with no previous history of concussion were precisely matched on self-reported ADHD (two subjects in each group). Matching was also attempted with self-reported learning problems (two subjects in the three or more concussions group and one subject in the no concussion group), the need for “special education” (one subject in the three or more concussions group and no subjects in the no concussion group), and repeated grades (three subjects in the three or more concussions group and two subjects in the no concussion group).

In addition to matching the above demographic and academic information, we also sought to match the participants on athletic variables. With the exception of one lacrosse player who was matched to a baseball player in the three or more group, we matched each of the athletes on the sport that they played (e.g., football, 30.8%; soccer, 23.1%; ice hockey, 15.4%; lacrosse, 15.4%; wrestling, 7.7%; and water polo, 3%). When possible, position played (e.g., forward in ice hockey, linebacker in football, goalkeeper in soccer, and weight class in wrestling) and school attended or the organization that they identified was also matched to controls.

### 2.2. Measures

ImPACT is a brief computer-administered neuropsychological test battery that consists of six individual test modules that measure aspects of cognitive functioning including attention, memory, reaction time, and processing speed. Each module contributes to the calculation of four composite scores, Verbal Memory, Visual Memory, Reaction Time, and Processing Speed.

- The Verbal Memory composite score represents the average percent correct for a word recognition paradigm, a symbol number match task, and a letter memory task with an accompanying interference task. These tests are conceptually similar to traditional verbal learning (word list) tasks and the auditory consonant trigrams test (i.e., the Brown-Peterson short-term memory paradigm), although the information is presented visually on the computer, not verbally by an examiner.
- The Visual Memory composite score is comprised of the average percent correct scores for two tasks; a recognition memory task that requires the discrimination of a series of abstract line drawings, and a memory task that requires the identification of a series of illuminated X's or O's after an intervening task (mouse clicking a number sequence from 25 to one). The first test taps immediate and delayed memory for visual designs and the second test measures short-term spatial memory (with an interference task).
- The Reaction Time composite score represents the average response time (in milliseconds) on a choice reaction time, a go/no-go task, and the previously mentioned symbol match task (which is similar to a traditional digit symbol task).
- The Processing Speed composite represents the weighted average of three tasks that are done as interference tasks for the memory paradigms.

In addition to the cognitive measures, ImPACT also contains a Postconcussion Symptom Scale that consists of 21 commonly reported symptoms (e.g., headache, dizziness, “fogginess”). The dependent measure is the total score derived from this 21-item scale.

The reliability [49–52] and validity [26, 53, 54] of the cognitive composite scores and the Postconcussion Symptom Scale [55–57], and the sensitivity of the battery to the acute effects of concussion [14, 15, 50, 58–62] have been examined in a number of studies. In three studies, the test-retest reliability of ImPACT over brief [49, 50] and long [52] retest intervals was considered adequate, and in one study stability was poor [51]. In regards to concurrent validity, the ImPACT composites measuring speed and reaction time showed medium correlations with the Symbol Digit Modalities Test, Trails B, and Digit Symbol [26, 53, 54], and the memory composites showed medium to large correlations with the Brief Visuospatial Memory Test [26, 53, 54].

## 3. Results

The athletes with previous concussions and the control subjects were compared on the four neuropsychological composite scores from ImPACT using multivariate analysis of variance (MANOVA) followed by univariate ANOVAs. Box's *M* Test was nonsignificant (*P* = .65), indicating that the covariance matrices were similar across the dependent variables. Levene's test was nonsignificant for all four composite scores, indicating that the assumption of homogeneity of variance was not violated. Across the two groups, however, there were some departures from normality on individual composite scores as assessed by the Kolmogorov-Smirnoff procedure. MANOVA and ANOVA tend to be quite robust to these violations of underlying general linear model assumptions. MANOVA, with the four cognitive composite scores as dependent variables and group membership as an independent variable, revealed no overall significant effect (*F*(4, 47) = 1.6, *P* = .19, observed power =  .46). The power for this analysis was low, increasing the risk for a type II statistical error. Therefore, exploratory one-way analyses of variance were conducted using Verbal Memory, Visual Memory, Reaction Time, Processing Speed, and Postconcussion Symptom composite scores as dependent variables and group membership as the independent variable. There was a significant effect for only the Verbal Memory composite (*P* = .028, Cohen's *d* = .63, medium effect size). The Visual Memory and Postconcussion Symptom composite scores were both not significant in the one-way analyses (*P* = .16 and *P* = .13, resp.), although there were small-medium effect sizes for both (*d* = .39 and *d* = .45, resp.). Performances on the Processing Speed and Reaction Time composites were very similar between groups. A summary of the athletes' performance on ImPACT, including age-adjusted percentile rank normative scores, is presented in Table 1.

## 4. Discussion

The results of this study were provocative but not persuasive. There was modest evidence that athletes with multiple concussions could have a lingering deficit in memory. Athletes with three or more previous concussions performed more poorly on the Verbal Memory composite score (Cohen's *d* = .63, medium effect) than athletes with fewer concussions. In a previous study, Iverson and colleagues reported a nonsignificant trend for athletes with three or more concussions to have worse preseason memory performance on ImPACT than those with no previous concussions (Cohen's *d* = .59, medium effect size), and recently concussed athletes with a history of multiple concussions had significantly greater decrements on the memory composite compared to concussed athletes with no injury history [34]. The present study, like several previous studies, is underpowered, which might explain why the medium effect sizes for Visual Memory and the total score from the Postconcussion Scale were not significant.

The literature to date regarding cumulative effects is not definitive. Some researchers have reported that groups of athletes with a history of multiple concussions perform more poorly on neuropsychological testing compared to those with fewer or no concussions [2, 39], whereas others have not [27, 31, 32]. Some researchers have reported that athletes with a history of multiple concussions report more subjective symptoms during preseason testing [2, 28, 34, 45], whereas others have not [39]. In the previously discussed large-scale study with jockeys, Wall and colleagues [39] emphasized that those individuals with two or more concussions versus a single past concussion performed more poorly on the Stroop Color-Word test, suggesting that “multiple concussions may interfere with executive skills, including response initiation/inhibition, divided attention, and concentration” (page 519). However, there was no significant difference between jockeys who were multiply concussed and those who were never concussed on the Stroop test (*d* = .22). Moreover, the jockeys with one or more past concussions actually performed significantly better on a test of attention and processing speed (digit symbol-coding) than jockeys with no previous concussions (*d* = .23, small effect size). Therefore, although the authors emphasized the possibility of an adverse lingering neurocognitive effect of multiple concussions, the results from that study could be interpreted as equivocal.

In the previous large-scale NCAA football study, Collins and colleagues [2] reported that “a history of concussion is significantly and independently associated with long-term deficits in the domains of executive functioning and speed of information processing” (page 968). Indeed, those players with a history of two or more concussions performed more poorly on Trails B (*d* = .41) and the Symbol Digit Modalities Test (*d* = .46). These findings appear to support their conclusion regarding long-term deficits. However, it is interesting to note that players with one previous concussion performed significantly better than players with no previous concussions on Digit Span (*P* < .03; *d* = .25, small effect), Symbol Digit Modalities Test (*P* < .03, *d* = .26), Trails A (*P* < .05, *d* = .23), and Trails B (*P* < .03, *d* = 26). The effect sizes, however, were very small. Nonetheless, a trend toward better neuropsychological performance in those with a history of concussion is difficult to understand and reconcile.

Belanger and colleagues [63] conducted a meta-analysis of the literature on lingering effects of multiple concussions. Eight studies met inclusion criteria, contributing 614 cases of multiple concussions and 926 control cases of a single concussion. There was no significant overall effect of multiple concussions on symptom reporting or neuropsychological test performance. However, using specific cognitive domains as a moderator, they reported that worse performance in both executive functions (*d* = .24; small effect) and delayed memory (*d* = .16; small effect) was associated with multiple concussions. As noted in the Introduction, there are numerous and important methodological differences and limitations in this literature. There is considerable heterogeneity in sample sizes, outcome measures, research design, and data analyses. These methodological differences and limitations make the literature challenging to interpret and to compare. Nonetheless, the lack of convergent evidence across studies and the meta-analysis suggests that lingering neurocognitive difficulties (a) are difficult to detect with standard neuropsychological testing and/or (b) might be present in only a subset of athletes and are thus obscured in group analyses.

The present study, unfortunately, is not immune to some of those same limitations. First, the relatively small sample sizes decreased the statistical power of the study. Small sample sizes of multiply concussed athletes have been a common methodological limitation in previous studies (e.g., [27–31, 34]). This limitation is difficult to overcome because of the difficulty identifying a large cohort of multiply concussed athletes. From a database of 786 subjects, we were able to extract only 26 with history of three or more injuries. Second, we were unable to define the severity of prior concussions. This limitation also is common in the literature (e.g., [9, 27, 32–39]). Third, we were unable to determine when the prior concussions occurred. This limitation, too, is common in the literature (e.g., [9, 22, 27, 28, 30, 32, 34–38]). Lastly, similar to past studies [2, 22, 27–29, 32, 34, 35, 38, 39, 41], we utilized a cross-sectional methodology (not longitudinal). Thus, clear causal inferences cannot be made.

Despite these aforementioned limitations, this study used a careful matching methodology. The rigor of this matching process minimized the likelihood that any differences found in neurocognitive performance are attributable to extraneous factors, such as age, education, self-reported ADHD, academic, or learning problems; sport; position played, and school or organization. Of course, more research is needed to better understand the extent to which there are lingering effects of multiple concussions, how the field best measures those effects, and how we use this information when making decisions regarding athletes' return to play.

## Figures and Tables

**Table 1:** Performance on ImPACT in athletes with 3 or more previous concussions and matched controls.

ImPACT scores	3 or more previous concussions		No previous concussion		*t*-test	Effect size
	Mean	SD	Mean	SD	(*P* value)	(Cohen's *d*)
*Raw scores*						
Verbal memory	86.4	8.1	92.1	10.0	5.14 (.028)	.63
Visual memory	77.1	13.3	82.3	13.1	1.99 (.16)	.39
Processing speed	41.7	7.5	41.3	8.2	0.21 (.88)	.04
Reaction time	0.55	0.08	0.54	0.08	0.56 (.46)	.21
Total symptoms	4.2	7.8	1.6	3.5	1.54 (.13)	.45

*Percentile ranks*						
Verbal memory	44.4	31.8	71.3	27.2	10.75 (.002)	.91
Visual memory	50.5	33.0	63.1	31.1	2.00 (.16)	.39
Processing speed	66.5	29.2	66.8	28.9	0.002 (.97)	.01
Reaction time	54.3	33.1	58.6	29.9	0.24 (.63)	.14

Note: SD: standard deviation. “Percentile ranks” refer to the age adjusted percentile ranks for each raw score from the ImPACT normative data. By convention, Cohen's effects sizes are interpreted as follows: small: 0.2, medium: 0.5, and large: 0.8.
